# Acute Promyelocytic Leukemia-like AML: Genetic Perspective and Clinical Implications

**DOI:** 10.3390/cancers16244192

**Published:** 2024-12-16

**Authors:** Luca Guarnera, Emiliano Fabiani, Giulia Falconi, Giorgia Silvestrini, Maria Luigia Catanoso, Mariadomenica Divona, Maria Teresa Voso

**Affiliations:** 1 PhD in Immunology, Molecular Medicine and Applied Biotechnology, Department of Biomedicine and Prevention, University of Rome Tor Vergata, 00133 Rome, Italy or luca.guarnera@students.uniroma2.eu (L.G.); or giorgia.silvestrini@alumni.uniroma2.eu (G.S.); 2 Department of Biomedicine and Prevention, University of Rome Tor Vergata, 00133 Rome, Italy; emiliano.fabiani@uniroma2.it (E.F.); or giulia.falconi@ptvonline.it (G.F.); or marialuigia.catanoso@opbg.net (M.L.C.); 3 UniCamillus-Saint Camillus International University of Health Sciences, 00131 Rome, Italy; 4 Policlinico Universitario Tor Vergata, 00133 Rome, Italy; mariadomenica.divona@ptvonline.it; 5 Neuro-Oncohematology Unit, Istituto di Ricovero e Cura a Carattere Scientifico (IRCCS), Fondazione Santa Lucia, 00142 Rome, Italy

**Keywords:** acute promyelocytic leukemia, APL, APL-like AMLs, atypical rearrangements, *RARA*, *RARB*, *RARG*, *PLZF::RARA*

## Abstract

Acute promyelocytic leukemia (APL) is a rare type of AML, characterized by the t(15;17) translocation and accounting for 8–15% of cases. The introduction of target therapies, such as all-trans retinoic acid (ATRA) and arsenic trioxide (ATO), radically changed the management of APL, making it the most curable AML subtype. However, a small percentage (estimated to be 2%) of AML presenting with APL-like morphology and/or immunophenotype lacks t(15;17). This rare APL-like AML group, whose first case was described in the early 1990s, now includes over 40 entities. These diseases present great heterogeneity in terms of genetic lesions, clinical presentation, sensitivity to targeted agents and chemotherapy, and prognosis. Furthermore, the diagnosis is very challenging. Thus, in this paper, we aim to comprehensively review the literature reports and studies addressing APL-like entities, investigate the biological mechanisms of leukemogenesis, evaluate the clinical characteristics, and discuss future lines of research and possible clinical approaches.

## Simple Summary

Acute myeloid leukemias (AML), mimicking acute promyelocytic leukemia (APL) features but lacking the canonical t(15;17) translocation, are rare but challenging entities, both on a diagnostic and clinical plan. From the first report, in the early 1990s, of an APL-like AML characterized by the t(11;17) translocation, several cases have been described, with significant heterogeneity in terms of presentation, sensitivity to treatments, and prognosis. In this review, we aim to describe APL-like entities reported to-date and discuss their biological background and clinical implications.

## 1. Introduction

Acute promyelocytic leukemia (APL) is a rare type of acute myeloid leukemia (AML), originally described in 1957, and accounting for 8–15% of cases (incidence of 0.12 per 100,000 person-years in Europe, with significant differences across countries) [1,2,3]. APL is caused, in the vast majority of cases, by the balanced t(15;17)(q24.1;q.2) translocation, which brings about the fusion of promyelocytic leukemia (*PML*) with retinoic acid receptor α (*RARA*) gene. This oncogene represses the transcription of *RARa* target genes and disrupts PML nuclear bodies, with subsequent impairment of differentiation, self-renewal, and response to DNA damage [4,5,6]. The natural history of APL is characterized by dismal prognosis due to the aggressive course of the disease, the resistance to chemotherapy, and the thrombo-hemorrhagic diathesis, responsible for a high rate of deaths within 30 days from diagnosis [7,8]. The introduction of the therapeutic armamentarium of drugs targeting both the pathologic fusion transcript and the leukemic niche, such as all-trans retinoic acid (ATRA), which displaces the repressor histone deacetylase, responsible for the *PML::RARA* blast differentiation impairment, and arsenic trioxide (ATO), which causes the degradation of blasts targeting PML, represented a major breakthrough in APL clinical management [9,10]. The seminal APL0406 trial, in fact, showed great efficacy of this chemo-free combination in standard-risk APL (presenting with less than 10 10^9^/L white blood cells) versus the classic AIDA (ATRA-idarubicin) chemotherapy [11,12], which remains the standard treatment for high-risk APL (WBC > 10 10^9^/L). In this setting, the APOLLO trial, testing the ATRA-ATO combination with low-dose chemotherapy vs. AIDA chemotherapy in high-risk patients, also showed promising results [13]. These clinical breakthroughs were confirmed in real-life settings: in the large HARMONY cohort, including more than 1400 APL patients deriving from both clinical trials and real-life scenarios, 91.7% and 83.8% survival rates were reached at 5 years in patients receiving ATRA-ATO and ATRA-chemotherapy, respectively [14,15]. Specific ATRA-ATO related short- and long-term side effects have been reported, including hyperleukocytosis and differentiation syndrome (DS), QT prolongation, liver and neurological toxicity [16,17]; these complications, though, are rarely life-threatening and easily manageable.

The APL-like spectrum includes AMLs with APL-like phenotypes, including translocations involving the loci on chromosome 15 or 17, but lacking the canonical t(15;17) accounts for 2% of AML presenting with APL morphology and/or immunophenotype [18].

APL presents, indeed, a specific flow cytometry profile, characterized by positivity for the CD33 and CD13 myeloid antigens, negativity for HLA-DR, and low frequency of CD34 expression [19,20]. Furthermore, CD2 and CD19 positivity has been correlated with microgranular morphology [21,22]. The phenotype of APL-like AML mimics that of classical APL; however, intriguingly, AMLs harboring *RARA* rearrangements may be CD56-positive, a feature not displayed by classical APL [23,24].

**Figure 1:**
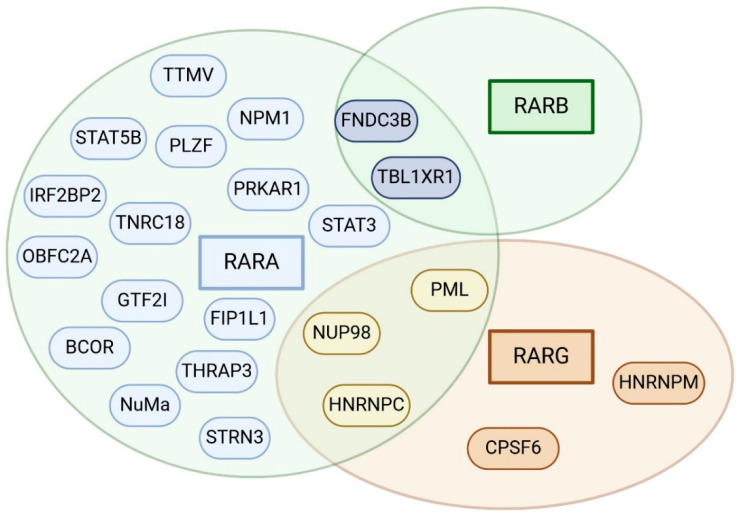
Overview of rearrangement partners of *RAR* genes. The color of partner genes matches the color of *RAR* genes with whom rearrangements were described. Some genes were described as partners of more than one component of *RAR* family and are shown in the overlapping areas between circles.

Despite the clinical characteristics shared with APL, these entities are characterized by variable thrombo-hemorrhagic diathesis and sensitivity to ATRA/ATO, with subsequent different incidence of differentiation syndrome (DS) and, overall, a dismal prognosis [18]. APL-like entities include translocations involving partners different from *PML*, *RARA*, or other genes from the *RAR* family, such as *RARB* and *RARG*; complex translocations (involving more than two partners); and translocations not involving *RAR* family (Figure 1) [18].

**Figure 2:**
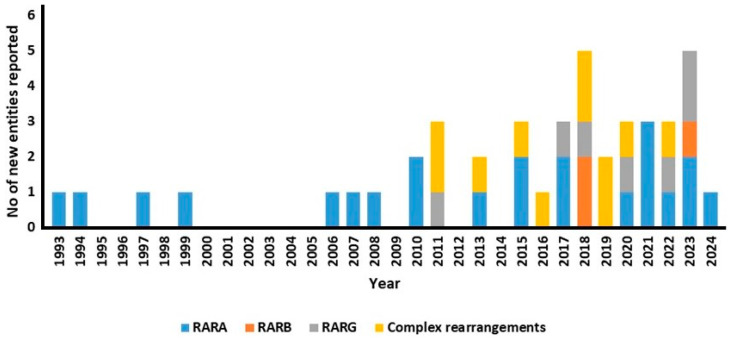
Histogram showing the number of reports on new fusion partners described in APL-like AMLs over time. AML: acute myeloid leukemia; APL: acute promyelocytic leukemia, No: number.

To date, 42 different APL-like entities involving *RAR* gene family members have been reported, whereas the nebula of AMLs resembling APL without the canonical genes involved is ill-defined. The raising awareness towards APL-like symptoms and the modern tools led to an increasing trend, over time, in the number of reports on new entities (Figure 2). Despite a deeper knowledge of the fusion partners and the biological background of these diseases, APL-like AMLs represent a tough diagnostic and clinical challenge. Thus, this review aims to summarize the evidence on these diseases, highlight the molecular processes of leukemogenesis, and explore the clinical implications.

## 2. Rearrangements Involving *RARA*

The most common translocations harbored by APL-like AMLs involve *RARA*. To date, 20 partners have been identified (Table 1), with the most common and studied *PLZF* [t(11;17)(11q23;q21)], also known as *ZBTB16*, which is estimated to account for 1% of all cases presenting with APL-like features [18,24,25,26,27]. Another *RARA* partner reported multiple times is *STAT5*, whose translocation [t(17;17)(q21;q21)] has been described in 21 patients since 1999, the year of the first report [24,28,29,30,31,32,33,34,35,36,37,38,39,40,41,42]. These two frequent rearrangements share resistance to differentiating agents ATRA and ATO and a dismal prognosis. In a worldwide multicenter collaboration, our group gathered several cases of APL-like AMLs, and those ATRA-resistant, harboring *ZBTB16-RARA* and *STAT5B-RARA* rearrangements, presented an overall survival (OS) and event-free survival (EFS) of 60.9 and 56.7% at 12 months, respectively. On the contrary, the three cases with *PRKAR1A-RARA*, *NuMA-RARA*, and *FIP1L1-RARA* rearrangements, treated with ATRA/chemotherapy (CHT) without hematopoietic stem cell transplantation (HSCT), were alive at 15–69 months from diagnosis [24]. The other translocations reported to date are shown in Table 1, along with the coagulopathy prevalence, sensitivity to treatment (both differentiating agents and chemotherapy), incidence of DS, and overall outcome.

As reported in the table, roughly half of the cases showed sensitivity to ATRA/ATO, and, in some cases [e.g., those bearing *FIP1L1::RARA* rearrangement, t(4;17)(q12;q21)], results on the efficacy of chemotherapy were conflicting, possibly due to different schedules, patients’ characteristics (e.g., performance status, age), or disease features (e.g., additional cytogenetic abnormalities or somatic mutations).

It is worth mentioning a recently described case, not reported in the table due to its complexity, with concurrent *STAT3::RARA* and *RARA::STAT5b* rearrangements, who presented with APL-like features. The patient was treated with ATRA and ATO, did not show signs of differentiation or clinical response, also to CHT, and died of intracranial hemorrhage [93].

The report of new variants paired with studies aiming at investigating the biology of these APL-like entities.

Chen and colleagues [70] first reported a *TBL1XR1::RARA* rearrangement [t(3;17)(q26;q21)] in a patient responding to ATO in combination with mitoxantrone as a salvage treatment after ATRA-CHT failure (the therapy was discontinued early due to pulmonary infection) [70]. Using mouse models harboring *TBL1XR1-RARA*, the authors showed an increased proliferative capacity of hematopoietic stem cells and a block in myeloid differentiation. The leukemic potential of this translocation was proven by the onset of an APL-like disease in 3 out of 15 mice transfected. Finally, the authors did not observe a survival advantage in mice treated with ATRA and/or ATO, but based on histone deacetylation phenotypes implied by bioinformatic analysis, inhibitors of histone deacetylase (HDACIs) were able to inhibit the proliferative capacity of leukemia cells and prolong survival [94]. In a subsequent paper, the authors transfected APL cell lines (HL-60 and U937) with either *TBL1XR1-RARA* mRNA or an empty vector as a control. *TBL1XR1-RARA* cells exhibited higher prevalence of apoptosis at both 24 and 48 h after ATO treatment when compared to controls. Furthermore, flow cytometry assay (CD11b and CD14 expression) and morphology observation showed time- and dose-dependent ATO-induced differentiation, higher in *TBL1XR1-RARA* when compared to controls [95]. All in all, despite conflicting results provided by in vitro experiments, in vivo drug testing, and the case reports available in the literature, it seems reasonable to treat patients harboring *TBL1XR1-RARA* rearrangement with a combination therapy including differentiating agents. The histone deacetylation profile, along with the in vitro efficacy of HDACIs, may suggest biologic commonalities with *PLZF-RARA* AML, discussed in the next chapter.

### 2.1. PLZF::RARA

As mentioned above, APL-like AMLs harboring *PLZF::RARA*, despite remaining an unmet medical need, have been thoroughly investigated, and the processes of leukemogenesis partially elucidated. Reciprocal translocation between *ZBTB16* on chromosome 11 and *RARA* genes on chromosome 17 [t(11;17)(q23;q21)] occurs, in fact, in 1–2% of APL-like cases [18]. Since its first description in the early 1990s [96], several cases have been reported, allowing a deeper knowledge of the clinical phenotype, characterized by resistance to ATRA and ATO differentiating agents, and of the pathogenic mechanisms underpinning the aggressive course. Immunofluorescence studies revealed that both PML-RARα and PLZF-RARα share a primarily diffuse nuclear pattern [97,98] with, though, profound differences in transcription repression mechanisms [99]. PLZF N-terminal POZ domain plays a pivotal role in DNA binding [100,101], allowing the recruiting of co-repressive factors such as SMRT, N-CoR, TRAC, and RIP13 [102,103]. These specific PLZF interactions, with subsequent transcriptional dysregulation and differentiation block, are responsible for the different disease phenotypes (chronic myeloid leukemia-like in mice models vs. classic APL phenotype in *PML::RARA* models) and the resistance to ATRA and ATO [104]. The observation of a histone deacetylation-mediated transcriptional impairment in *PLZF::RARA* models [104] led to the investigation, by independent research groups, of HDACIs [e.g., trichostatin A (TSA), phenylbutyrate, suberanilohydroxamic acid (SAHA)]. These drugs induced differentiation and remission in in vitro and in vivo models but were not implemented in clinical practice [103,105].

Other dysregulated pathways and potential therapeutic targets detected in PLZF-RARα leukemia (which, however, were not further investigated) included upregulation of *CRABPI* (cellular retinoic acid binding protein 1 [106]), *EYA2* (EYA transcriptional coactivator and phosphatase 2 [107]), and *USP37* (ubiquitin-specific peptidase 37 [108]).

Our group characterized the mutational profiles of seven *PLZF::RARA* leukemia cases, collected through a worldwide collaboration, and compared them with a cohort of canonical APLs and non-APL AML [27]. The number of mutations per patient was higher in *PLZF-RARA* vs. APL and lower than that of the non-APL AML group (1.71 vs. 0.89 vs. 2.86). Remarkably, using whole exome sequencing or mutational profiling of a comprehensive 409 tumor suppressors and oncogenes panel, we found a high incidence of *ARID1A* mutations (five cases, 71%) and mutations in *ARID2* and *SMARCA4*, other tumor suppressor genes belonging to the SWI/SNF chromatin remodeling complex (one case each, 14%) in *PLZF-RARA AML* [105].

Remarkably, an *ARID1A* mutation has been recently described in a patient with APL-like AML harboring *TTMV*::*RARA* rearrangement, possibly suggesting a common mechanism of leukemogenesis [47].

The SWI/SNF complex belongs to three main subfamilies: canonical BAF (cBAF), polybromo-associated BAF (PBAF), and the GLTSCR1- or GLTSCR1L-containing and BRD9-containing (GBAF) complex, also known as non-canonical BAF (ncBAF). The complex localizes at sites marked by histone H3 lysine 27 acetylation (H3K27ac) and fosters transcription by cooperating with transcription factors and antagonizing EZH2 [Enhancer of Zeste Homolog 2, a functional enzymatic component of the Polycomb Repressive Complex 2 (PRC2)]-mediated trimethylation of H3K27 (H3K27me3) [109].

In this line, a recent paper, using single-cell multi-omics, identified a driver role of EZH2 in ATRA resistance. Intriguingly, the simple inhibition of EZH2 (with GSK, an S-adenosyl-methionine competitor that inhibits EZH2 methyltransferase activity) did not impact disease progression and blast differentiation, whereas the EZH2 degrader MS1943 erased the global level of H3K27me3, decreased *PLZF-RARα* expression at both mRNA and protein levels, induced cell differentiation, and reduced cell viability [110].

### 2.2. TTMV::RARA

In 2021, Astolfi and colleagues [43] reported the first known case of a pediatric APL-like AML lacking the canonical translocation, characterized by the integration of the torque teno mini virus (TTMV) into the *RARA* locus. The patient was a 6-year-old child presenting with mild trilineage cytopenia, consumptive coagulopathy, and atypical promyelocytes packed with numerous azurophilic granules at peripheral blood smear examination. The patient was treated with ATRA + CHT and achieved CR. Unfortunately, 8 months later, a relapse occurred. Whole-transcriptome sequencing detected an aberrant expression of a portion of *RARA* intron 2, linked to a fusion transcript involving the integration of 209 nucleotides upstream of *RARA* exon 3. The integrated sequence revealed a significant alignment to TTMV and showed the conserved domain of the Torque Teno Open Reading Frame 2 (ORF2) superfamily. The patient was treated with an ATRA/ATO combination and achieved morphologic CR. This achievement was consolidated by HSCT, and at the time of the case report, the patient was alive [43]. This finding prompted the authors to examine the transcriptome analyses of previous pediatric patients diagnosed with cytogenetically normal AML and found another case of a 3-year-old patient with an integrated TTMV sequence, highly homologous to that of the index case. Of note, the expressed sequence was longer (328 base pairs in this last patient) [43].

The discovery of these two cases paved the way for other researchers to investigate these integrated sequences in APL-like AML, lacking the *PML::RARA* fusion gene.

In 2022, Chen et al. [44], through a retrospective transcriptome analysis of patients with AML, identified the third case of *TTMV::RARA in* a 3-year-old child with hyperleukocytosis and bone marrow (BM) smears infiltrated by 73.6% of hyper-granular promyelocytes. After a few days of ATRA treatment, CHT was administered with the achievement of CR. A month later, the patient relapsed and underwent CHT with subsequent HSCT. Unfortunately, the patient relapsed again and died 50 days later [44]. Intriguingly, the principal component analysis, including APL, AML, and healthy subjects, revealed that the above-described case was separated from other AML cases and clustered adjacent to the APL cohort [44].

Sala-Torra and colleagues [45], in 2022, reported another *TTMV::RARA* rearranged case of a 39-year-old man presenting with thrombocytopenia, hemorrhagic diathesis, and diffuse intravascular coagulation; peripheral blood analysis revealed APL-like blasts, and the patients underwent a standard cytarabine–daunorubicin chemotherapy regimen without benefit. As reinduction, the patients underwent chemotherapy with mitoxantrone, etoposide, and cytarabine with decitabine and venetoclax with a response [45].

In 2024, Wang Z. et al. [46] described a case of a 15-year-old patient presenting with hyperleukocytosis, anemia, thrombocytopenia, and severe alteration of coagulation parameters. After a short-term CR, obtained with a ATRA-chemotherapy regimen followed by ATRA-ATO consolidation, the patient experienced multiple relapses, also involving central nervous system infiltration [46]. Wang L. et al. [47] reported a similar case of a 9-year-old patient presenting with hyperleukocytosis, anemia, and thrombocytopenia. Also in this case, central nervous system infiltration was documented. The patient was treated with venetoclax and low-dose cytarabine, showing resistance. CR was finally obtained with venetoclax–azacitidine, followed by homoharringtonine–cytarabine consolidation and intrathecal injection of methotrexate–cytarabine–dexamethasone. The patient underwent HSCT but, after 13 months, relapsed. Currently, after several courses of chemotherapy and targeted therapy, the physicians are searching for suitable donors for a potential second HSCT [47].

These reports broadened our knowledge of this entity, characterized by an aggressive course and unclear response to differentiating agents and CHT, with a high rate of treatment failure and relapse (see Table 1). Biologically, these cases present heterogeneous fusion transcript lengths and a challenging diagnosis. In this line, a recently published article by Tsai and colleagues shed light on the molecular recognition and monitoring of *TTMV::RARA* AMLs. Using DNA- or RNA-based custom NGS assays, the authors identified four cases without known cytogenetic/molecular drivers. Remarkably, one of these cases was identified prospectively, allowing for a measurable residual disease monitoring of the fusion transcript [48].

## 3. Non-RARA Rearrangements

Rearrangements involving partners other than *RARA* include *RARB*, *RARG*, or complex rearrangements with more than one partner. Involvement of genes not belonging to the *RAR* family has also been described in APL-like AML.

### 3.1. RARB

Few rearrangements involving *RARB* have been reported so far. The most common is *TBLXR1::RARB*, reported in seven pediatric cases (median age: 3 years, range: 1–5 years) [111,112,113,114,115]. *TBLXR1* encodes for a transcriptional regulatory protein interacting with the NCoR (nuclear receptor corepressor)/SMRT (silencing mediator of retinoic acid and thyroid hormone receptors) complex. This results in the stabilization of the complex on the chromatin, through histone H2 and H4 interaction, acting as a transcriptional corepressor and mediating its ubiquitination and degradation [116,117,118]. *TBLXR1* regulation activity also involves NF-κB and the WNT signaling pathways [116].

The cases observed were resistant to ATRA treatment but showed good responses to chemotherapy, with five out of seven patients alive at the time of the case reports at a median follow-up of 63 months. Of note, *TBLXR1* partners also with *RARA* [70,71,72,73], and reported cases showed sensitivity to combination therapy.

Two other *RARB*-rearranged AMLs, both sensitive to chemotherapy and with good outcomes, have been reported (Table 2). *FNDC3B*, fused with *RARB* in a pediatric case of APL-like AML, has also been described as a partner of *RARA* [86].

### 3.2. Rearrangements Involving RARG

Over 50 cases of APL-like AMLs involving *RARG* have been reported. The most common partner is *CPSF6* (cleavage and polyadenylation specific factor 6), a subunit of cleavage factor I, which is involved in messenger RNA precursor 3′-end processing [120]. AML driven by *CPSF6::RARG* fusion gene, due to translocation t(12;12)(q13;q15) are characterized by resistance to ATRA and ATO, with variable sensitivity to CHT. These features are shared with *NUP98::RARG*-driven AML [t(11;12)(p15;q13)], the second most common rearrangement involving *RARG.* Less frequently reported partner genes are *HNRNPC*, *PML*, and *NRNPM* (Table 3).

A large, multicenter study by Zhu et al. [121] gathered data from 34 *RARG*-rearranged AML. None of the patients responded to ATRA and/or ATO. The rate of bleeding and ecchymosis was as high as 55%, and 10 patients (39%) died within 45 days after diagnosis. The prognosis was dismal, with an estimated 2-year cumulative incidence of relapse, EFS, and OS of 68.7%, 26.7%, and 33.5%, respectively. Finally, the authors, elaborating transcriptome data from 201 patients with AML using unsupervised hierarchical clustering, found that 81.8% of the *RARG* fusion samples clustered together, suggesting a specific molecular subtype [121].

Of note, in a case report describing an AML with *NUP98::RARG* rearrangement, Wu et al. observed a mutation in the *ARID1B* gene [122], suggesting a role of the SWI/SNF complex also in RARG-rearranged APL-like AMLs.

**Table 3:** Genetic features and clinical characteristics of APL-like entities harboring translocation involving RARG.

Fusion Genes	Cases (N)	Translocation	Coagulopathy	ATRA	ATO	CHT	Combo	DS	OS, Median FU (Range)	Ref.
*CPSF6::RARG*	26	t(12;12) (q13;q15	6 (85.7%) 19 ND	R	R	S/R	ND	N	10 mo (1–33); 5 alive; 4 dead; 17 ND	[26,112,121,123,124,125,126]
*NUP98::RARG*	23	t(11;12) (p15;q13)	5 (62.5%), 15 ND	R	R	R/S	ND	2 (18%)	20 mo (0–32); 6 alive; 5 dead; 12 ND	[121,122,127,128,129,130,131,132,133,134,135,136]
*HNRNPC::RARG*	3	t(12;19) (q13;q13.1)	1 (50%)	R	R	S/R	ND	0	11.5 mo (10–13); 2 dead, 1 ND	[121,137]
*PML::RARG*	2	t(12;15) (q13;q22)	0	R	ND	S	ND	0	case 1: CR, ND on FU; case 2: ND	[121]
*HNRNPM::RARG*	1	Cryptic	1 (100%)	ND	ND	R	ND	ND	10 mo, alive	[138]

Part of these data and the table layout have been adapted from Front Oncol 2022 [18] and updated with recently identified new cases. Data on the prevalence of coagulopathy derives from laboratory and/or clinical data provided by original sources. Translocations not shown by conventional karyotype/G-banded karyotype were considered cryptic. ATO: arsenic trioxide; ATRA: all-trans retinoic acid; chemo: chemotherapy; CHT: chemotherapy; Combo: ATRA+chemotherapy; CR: complete response; DS: differentiation syndrome; FU: follow-up; mo: months; N: number; ND: no data; OS: overall survival; R: resistant; Ref: reference; S: sensitive.

### 3.3. Complex Rearrangements

Anecdotal cases of three- or four-way rearrangements involving *RARA* or *RARG* have been reported (Table 4). The *PML::RARA* fusion gene was detected in most of them, with features and outcomes similar to canonical APL. These entities may be thus included in the group of APL presenting with additional cytogenetic abnormalities (10–30% of cases), whose prognosis is controversial. Six studies addressed this issue in the pre-ATO era: three of them showed inferior outcomes of patients with additional karyotype abnormalities vs. those with isolated t(15;17), while the other three did not observe significant differences [50,139,140,141,142,143]. Recently, Epstein-Peterson and colleagues [144] performed a pooled analysis of exclusively ATO-treated patients at a single academic institution and observed inferior EFS in patients harboring a complex karyotype but not for those harboring additional cytogenetic abnormalities [144].

### 3.4. Rearrangements Not Involving RAR Family

AML presenting with APL-like morphology and immunophenotype have been described, in particular *NPM1*-mutated and *KMT2a*-rearranged AML [156,157,158]. In particular, Fang and colleagues, comparing 47 APL, 26 *NPM1*-mutated AML, and 12 *KMT2A*-rearranged AML with an APL-like immunophenotype, found that the latter mimics hypogranular APL blasts (low side scatter) showing, though, lower expression of CD2 and CD34; furthermore, *NPM1*-mutated cases presented lower expression of CD13 and CD64, and *KMT2A*-rearranged cases lower expression of MPO [158,159]. The APL-like flow cytometry profile was reported in 30–50% of *NPM1*-mutated AML [160,161] (more common in the case of co-mutations of *TET2/IDH1/IDH2* genes [160]) and 8% of *KMT2A*-rearranged AML [162].

In a similar fashion, the coagulation disorders, common in APL, are observed in other AML phenotypes, including both bleeding (e.g., *KMT2a*-rearranged AML [163,164]) and thrombotic events (reported in up to 15% of not-APL AMLs [7,165,166]).

However, Zhao et al. [112] and Borkovskaia et al. [114] reported pediatric APL-like patients with rearrangements not involving the *RAR* gene family (Table 5).

Zhao et al. [112] compared, in a cohort of pediatric patients, 77 cases with APL with 18 APL-like patients without *RARA* rearrangements (including three cases harboring *CPSF6::RARG* and two cases harboring *TBL1XR1-RARB* and non-*RAR* rearrangements, as reported in Table 5). APL-like cases showed significant differences both in mutational profile (enrichment in *NPM1* and *TP53* mutations), clinical features (younger age at presentation), and outcomes (inferior OS and EFS) [112].

Borkovskaia and colleagues [114] reported a single-center experience of seven pediatric AML cases with APL features. One case harbored a *TBLXR1::RARB* fusion gene, one patient had a *KMT2A::SEPT6* fusion gene, and in five cases no fusion genes were detected. All patients (with the exception of one case, not presenting a fusion gene) were treated with ATRA+CHT combination therapy with a good response rate. When compared to APL cases, no difference in OS was detected. Of note, a trend toward lower EFS in APL-like cases was observed (*p* = 0.1) [114].

## 4. Management and Future Directions

APL-like AMLs represent a tough diagnostic and therapeutic challenge.

A strong suspicion of APL should prompt the early administration of ATRA and *PML-RARA* rearrangement investigation. In case of negativity, further investigations, such as RT-PCR for the most common *RARA* recurrent translocations, are warranted, together with conventional karyotyping.

Last advances in APL-like AMLs brought forward the knowledge on the biology of these diseases, possibly magnifying their biologic complexity. In some cases, complex chimeric transcripts have been detected, including fusion-adjacent genes located on the same chromosome [168]. In a similar fashion, not only a 5′ fusion partner but also a 3′ fusion partner, most consisting of transposons, have also been described in APL-like AMLs. The additional fusion partner may explain the different sensitivity to ATRA of these APL-like AML [169]. The potential implications of these findings are yet to be validated.

On a clinical level, lacking specific guidelines, the choice of treatment should be guided by previous reports available in the literature. Of note, several cases have been successfully treated with less-intensive therapies (hypomethylating agents and/or venetoclax) [47,49,81,89,135,170]. Considering the high rate of relapse observed in these patients, the autologous and/or HSCT option, which has a key role in relapsed/refractory APL, should be considered [171,172].

## Figures and Tables

**Table 1:** Genetic features and clinical characteristics of APL-like AMLs harboring translocations involving RARA.

Fusion Genes	Cases (N)	Translocation	Coagulopathy	ATRA	ATO	CHT	Combo	DS	OS, Median FU (Range)	Ref.
*PLZF::RARA*	1% of total	t(11;17) (11q23;q21)	40–55%	R	R	S	S	NR	25 mo; 40% alive	[19,20,22,38]
*STAT5B::RARA*	21	t(17;17)(q21;q21)	8 (47%), 10 ND	R	R	S	S	0	15 mo (0–53); 8 alive; 7 dead; 3 ND	[24,28,29,30,31,32,33,34,35,36,37,38,39,40,41,42]
*TTMV::RARA*	12	Cryptic	4 (57%), 5 ND	S/R	S/R	S/R	S	0	35 mo (4–56); 5 alive, 2 dead, 2 relapse, no data on FU, 1 CR ND on FU	[43,44,45,46,47,48,49]
*NPM1::RARA*	11	t(5;17)(5q35;q21)	2 (18%), 8 ND	S	ND	S	S	0, 3 ND	18 mo (0–46); 7 alive; 2 dead; 2 CR, ND on FU	[25,50,51,52,53,54,55,56]
*IRF2BP2::RARA*	7	t(1;17)(q42;q21)	2 (28.6%)	S	ND	ND	S	0	17 mo (1–50); 3 alive; 3 dead; 1 ND	[57,58,59,60,61,62]
*FIP1L1::RARA*	7	t(4;17)(q12;q21)	0, 1 ND	S	S	S/R	S/R	2 (29%)	6 mo (0–20); 4 alive; 3 dead	[63,64,65,66,67,68,69]
*TBL1XR1::RARA*	4	t(3;17)(q26;q21)	0, 2 ND	ND	ND	ND	S	0	5.5 mo (0–11); 2 dead; 1 CR no data on FU; 1 ND	[70,71,72,73]
*BCOR::RARA*	3	t(X;17)(p11;q21)	1 (33%)	S	R	S	S	0	26 mo (9–41); 3 alive	[74,75,76]
*STAT3::RARA*	2	t(17;17)(q21;q21)	ND	R	R	ND	R	0	20 mo (7–33); 2 dead	[77]
*PRKAR1A::RARA*	2	t(17;17)(q21;q24)	1 (50%)	ND	ND	ND	S	0	case 1: 24 mo, alive; case 2: CR, ND on FU	[78,79]
*TNRC18::RARA*	2	ND	1 (50%)	R	R	S	ND	0	7.5 mo (6–9); 2 alive	[80,81]
*HNRNPC::RARA*	2	t(14;17)(q11;q21)	0, 1 ND	R	ND	S	ND	0	7.5 mo (3–12); 1 alive, 1 dead	[82,83]
*OBFC2A::RARA*	1	t(2;17)(q32;q21)	0	ND	ND	S	S	0	15 mo, alive	[84]
*GTF2I::RARA*	1	t(7;17)(q11;q21)	1 (100%)	R	R	R	R	0	5 mo, dead	[85]
*FNDC3B::RARA*	1	t(3;17)(q26;q21)	1 (100%)	ND	ND	S	ND	1 (100%)	CR, ND on FU	[86]
*NUP98::RARA*	1	ND	1 (100%)	ND	ND	S	ND	0	44 mo, alive	[87]
*X::RARA*	1	t(X;17)(q28;q12)	ND	ND	ND	S	R	0	23 mo, dead	[88]
*RARA::THRAP3*	1	Cryptic	1	R	R	ND	ND	0	CR, ND on FU	[89]
*NuMa::RARA*	1	t(11;17) (11q23;q21)	ND	S	ND	ND	ND	0	38 mo, alive	[90]
*NAB2::RARA*	1	t(12;17)(q13;q21)	1	S	S	ND	ND	0	CR, ND on FU	[91]
*STRN3::RARA*	1	der(14)t(14,17) (q12;q21) dup(17)(q21q25)	0	S	S	ND	ND	0	12 mo, dead	[92]

Part of these data and the table layout have been adapted from Front Oncol 2022 [18] and updated with recently identified new cases. Data on the prevalence of coagulopathy derives from laboratory and/or clinical data provided by original sources. Translocations not shown by conventional karyotype/G-banded karyotype were considered cryptic. ATO: arsenic trioxide; ATRA: all-trans retinoic acid; chemo: chemotherapy; CHT: chemotherapy; Combo: ATRA+chemotherapy; CR: complete response; DS: differentiation syndrome; FU: follow-up; mo: months; N: number; ND: no data; NR: not reported; OS: overall survival; R: resistant; Ref: reference; S: sensitive.

**Table 2:** Genetic features and clinical characteristics of APL-like entities harboring RARB rearrangements.

Fusion Genes	Cases (N)	Translocation	Coagulopathy	ATRA	ATO	CHT	Combo	DS	OS, Median FU (Range)	Ref.
*TBLXR1::RARB*	7	Cryptic	1 (17%), 5 ND	R	ND	S	ND	ND	63 mo (23–108); 5 alive; 2 ND	[111,112,113,114,115]
*FNDC3B::RARB*	1	Cryptic	1	R	ND	S	ND	0	12 mo, alive	[119]
*X::RARB*	1	t(X;3)(q28;q21)	ND	R	ND	S	ND	ND	31 mo, alive	[113]

Part of these data and the table layout have been adapted from Front Oncol 2022 [18] and updated with recently identified new cases. Data on the prevalence of coagulopathy derives from laboratory and/or clinical data provided by original sources. Translocations not shown by conventional karyotype/G-banded karyotype were considered cryptic. ATO: arsenic trioxide; ATRA: all-trans retinoic acid; chemo: chemotherapy; CHT: chemotherapy; Combo: ATRA+chemotherapy; CR: complete response; DS: differentiation syndrome; mo: months; N: number; ND: no data; OS: overall survival; R: resistant; Ref: reference; S: sensitive.

**Table 4:** Genetic features and clinical characteristics of APL-like entities harboring translocations involving more than two partners, including one RAR gene family member.

Fusion Genes	Cases (N)	Translocation	Coagulopathy	ATRA	ATO	CHT	Combo	DS	OS, Median FU	Ref.
*TFG::RARA*	1	t(3;14;17)(q12;q11;q21)	0	S	ND	ND	S	0	3 mo, alive	[145]
*PML::RARA*	1	t(12;17;15)(p13;q21;q22)	ND	S	ND	ND	ND	0	CR, ND on FU	[146]
*PML::RARA*	1	t(12;15;17)(q24;q24;q11)	ND	ND	ND	ND	S	0	4 mo, alive	[147]
*PML::RARA*	1	t(1;17;15)(q21;q21;q24)	1 (100%)	ND	ND	ND	S	1 (100%)	48 mo, alive	[148]
*PML::RARA*	1	t(6;17;15)(p21;q21;q22)	1(100%)	S	S	ND	ND	0	60 mo, alive	[149]
*PML::RARA*	1	t(3;17;15)(q25;q21;q24)	1 (100%)	ND	ND	S	ND	0	4 mo, alive	[150]
*PML::RARA*	1	t(5;17;15;20) (q33;q12;q22;q11.2)	1 (100%)	ND	ND	ND	S	0	CR, ND on FU	[151]
*PML::RARA*	1	t(9;17;15;12;15) (q34;q21;q24;q13;q26.1)	1 (100%)	ND	ND	ND	S	1 (100%)	CR, ND on FU	[152]
*NPM1::RARG::NPM1*	1	ND	0	R	R	ND	ND	0	8 mo, dead	[153]
*PML-ADAMTS17-RARA*	1	ins(15;17) (q22;q21q25) inv(15)(q22q24)	1 (100%)	ND	ND	ND	S	1 (100%)	CR, ND on FU	[154]
*RARA-SNX15*	1	t(11;17;15) (q13;q21.2;q24.1)	1 (100%)	ND	ND	ND	S	1 (100%)	CR, ND on FU	[155]

Part of these data and the table layout have been adapted from Front Oncol 2022 [18] and updated with recently identified new cases. Data on the prevalence of coagulopathy derives from laboratory and/or clinical data provided by original sources. ATO: arsenic trioxide; ATRA: all-trans retinoic acid; chemo: chemotherapy; CHT: chemotherapy; Combo: ATRA+chemotherapy; CR: complete response; DS: differentiation syndrome; FU: follow-up; mo: months; N: number; ND: no data; OS: overall survival; R: resistant; Ref: reference; S: sensitive.

**Table 5:** Genetic features and clinical characteristics of APL-like AML harboring translocations not involving RAR gene family members.

Fusion Genes	Cases (N)	Translocation	Coagulopathy	ATRA	ATO	CHT	Combo	DS	OS, Median FU (Range)	Ref
*ELL::MLL/* *MLL::ELL*	2	t(11;19)(q23;p13.3)	1 (50%), 1 ND	ND	ND	ND	S	ND	170 mo, alive; 1 ND	[112,167]
*MLL::AF1Q*	1	t(1;11)(q21;q23)	ND	ND	ND	ND	S	ND	34 mo, alive	[112]
*RPRD2::MLL*	1	t(1;11)(q21;q23)	ND	ND	ND	ND	S	ND	34 mo, alive	[112]
*NPM1::CCDC28A*	1	ND	ND	ND	ND	ND	S	ND	54 mo, alive	[112]
*TBC1D15::RAB21*	1	ND	ND	ND	ND	ND	S	ND	56 mo, alive	[112]
*KMT2A::SEPT6*	1	ins(X;11)(q24;q14q25)	ND	ND	ND	ND	S	ND	30 mo, dead	[114]

Part of these data and the table layout have been adapted from Front Oncol. 2022 [18] and enriched with new cases identified recently. Coagulopathy was assessed based on laboratories and/or clinical data provided by original sources. ATO: arsenic trioxide; ATRA: all-trans retinoic acid; chemo: chemotherapy; CHT: chemotherapy; Combo: ATRA+chemotherapy; CR: complete response; DS: differentiation syndrome; mo: months; N: number; ND: no data; OS: overall survival; R: resistant; Ref: reference; S: sensitive.

## Abbreviations

AML	acute myeloid leukemia
APL	acute promyelocytic leukemia
ATO	arsenic trioxide
ATRA	all-trans retinoic acid
cBAF	canonical BAF
CHT	chemotherapy
CR	complete remission
CRABPI	cellular retinoic acid binding protein 1
DS	differentiation syndrome
EFS	event-free survival
EYA2	EYA transcriptional coactivator and phosphatase 2
EZH2	Enhancer of Zeste Homolog 2
FU	follow-up
GBAF	GLTSCR1L-containing and BRD9-containing
HSCT	hematopoietic stem cell transplantation
Mo	months
ncBAF	non-canonical BAF
NCoR	nuclear receptor corepressor
ND	no data
No	number
NR	not reported
ORF	open reading frame
OS	overall survival
PBAF	polybromo-associated BAF
PML	promyelocytic leukemia
R	resistant
RAR	retinoic acid receptor
Ref	reference
S	sensitive
SAHA	suberanilohydroxamic acid
SMRT	silencing mediator of retinoic acid and thyroid hormone receptors
TSA	trichostatin A
TTMV	teno mini virus
USP37	ubiquitin-specific peptidase 37
